# Epigallocatechin-3-Gallate Induces Apoptosis as a TRAIL Sensitizer via Activation of Caspase 8 and Death Receptor 5 in Human Colon Cancer Cells

**DOI:** 10.3390/biomedicines8040084

**Published:** 2020-04-09

**Authors:** Oh Sung Kwon, Ji Hoon Jung, Eun Ah Shin, Ji Eon Park, Woon Yi Park, Sung-Hoon Kim

**Affiliations:** College of Korean Medicine, Kyung Hee University, Seoul 02447, Korea; woorihanul@gmail.com (O.S.K.); johnsperfume@gmail.com (J.H.J.); eunah1008@khu.ac.kr (E.A.S.); wdnk77@naver.com (J.E.P.); wy1319@naver.com (W.Y.P.)

**Keywords:** EGCG, TRAIL, colon cancer, caspase, DR5, apoptosis

## Abstract

Though epigallocatechin-3-gallate (EGCG), a major compound of green tea, has anti-diabetes, anti-obesity, anti-inflammatory, and antitumor effects, the underlying antitumor molecular mechanism of EGCG was not fully understood so far. Here the sensitizing effect of EGCG to tumor-necrosis-factor-related apoptosis-inducing ligand (TRAIL) was examined in colorectal cancers. Cotreatment of EGCG and TRAIL synergistically enhanced cytotoxicity and sub G1 accumulation, increased the number of terminal deoxynucleotidyl transferase-dT-mediated dUTP nick end labelling (TUNEL)-positive cells in SW480 and HCT116 cells. Furthermore, this cotreatment promoted the cleavages of poly (adenosine diphosphate-ribose) polymerase (PARP) and induced caspase 8 activation compared to TRAIL or EGCG alone in SW480 and HCT116 cells. Of note, cotreatment of EGCG and TRAIL increased the expression of death receptor 5 (DR5) at protein and mRNA levels and also DR5 cell surface level in colon cancer cells. Conversely, depletion of DR5 reduced the apoptotic activity of cotreatment of EGCG and TRAIL to increase cytotoxicity, sub-G1 population and PARP cleavages in colon cancer cells. Overall, our findings provide evidence that EGCG can be a sensitizer of TRAIL via DR5 and caspase 8 mediated apoptosis in colorectal cancer cells.

## 1. Introduction

Though about 70%–80% of colorectal cancer (CRC) patients have been subjected to surgical resection worldwide, approximately 40% of them have metastases or recurrence and eventually die [1]. Furthermore, though anti-cancer agents including tumor-necrosis-factor-related apoptosis-inducing ligand (TRAIL) [2] and 5-Fluorouracil [3] have been used for treatment of CRCs, their chemo-resistance and side effects have become hot issues to be overcome. Thus, recently, herbal extracts and their natural compounds are of interest for their reducing side effects and sensitizing effects to anticancer agents including TRAIL [4].

It is well documented that TRAIL binds to DR5 (TRAIL-R2), DR4 (TRAIL Receptor-1) and three decoy receptors such as Osteoprotegerin (TRAIL-R5), DcR2 (TRAIL-R4) and DcR1 (TRAIL-R3) with a truncated cytoplasmic death domain [5]. Additionally, TRAIL is well known to induce apoptosis in breast [6], prostate [7], lung [8] and renal cancers [9,10] as a potent anticancer agent. Hence, emerging evidence suggests targeting TRAIL receptor can be a good strategy for cancer therapy including chemoresistance [11,12].

Accumulating evidences reveal that the anti-cancer effect of green tea is exerted mainly by its polyphenolic compounds [13,14]. The major catechin derivatives in leaves of green tea are comprised of eight phenolic flavonoid constituents, such as catechin, epicatechin, gallocatechin, epigallocatechin, catechin gallate, epicatechin gallate, gallocatechin gallate, and epigallocatechin gallate (EGCG) [15]. EGCG [16,17] was reported to show antitumor effects in several cancers [18]. However, its anti-tumor mechanisms still remain unclear in colon cancer cells to date.

Hence, in the current study, the sensitizing potential of EGCG was evaluated in TRAIL induced apoptosis in highly aggressive SW480 and HCT116 colon cancer cells.

## 2. Materials and Methods

### 2.1. Chemicals and Reagents

Epigallocatechin-3-gallate (EGCG) (molecular weight: 458.37), TRAIL, 3-(4,5-dimethylthiazol-2-yl)-2,5-diphenyltetrazolium bromide (MTT) and phosphatase inhibitors were purchased from Sigma (Sigma, St. Louis, MO, USA). Specific antibodies for DR5, DR4 (Santa Cruz Biotechnology, CA, USA) and poly (adenosine diphosphate-ribose) polymerase (PARP), Cleaved PARP, caspase 8 (Cell Signaling, Beverly, MA, USA) and β-actin (Sigma Aldrich Co., St. Louis, MO, USA) were purchased for Western blot analysis. Terminal deoxynucleotidyl transferase-dT-mediated dUTP nick end labelling (TUNEL) kit and protease inhibitors were bought from Roche (Roche Molecular Biochemicals, Mannheim, Germany).

### 2.2. Cell Culture

HT29 (ATCC® HTB-38™), HCT116 (ATCC**^®^** CCL-247™) and SW480 (ATCC**^®^** HTB-228™) colon cancer cells were obtained from American Type Culture Collection (ATCC, Manassas, VA, USA). Cells were cultured in Roswell Park Memorial Institute medium (RPMI) supplemented with 1% antibiotic (Welgene, Gyeongsan, Korea) and 10% fetal bovine serum (FBS).

### 2.3. MTT Assay

Cytotoxic effect of EGCG and/or TRAIL in SW480 and HCT116 cells was evaluated by using MTT assay according to the manufacturer’s instruction. In brief, the cells (1 × 10^4^ cells per well) were distributed onto a 96-well microplate and treated with various concentrations of EGCG (0, 20, 40, 60 μM) and/or TRAIL (0, 25, 50 ng) for 24 h. The cells were incubated with MTT (1 mg/mL) for 2 h, and then were exposed to MTT lysis solution overnight. Then, optical density was measured using a microplate reader (Molecular Devices Co., Silicon Valley, CA, USA) at 570 nm. Cell viability was calculated as a percentage of viable cells in EGCG and/or TRAIL treated group versus untreated control.

### 2.4. TUNEL Assay

The DeadEnd™ Fluorometric TUNEL system kit was used for detecting cell death, according to the manufacturer’s instructions. In brief, SW480 or HCT116 cells were treated with EGCG and/or TRAIL for 24 h and then washed with cold PBS. The cells were fixed with 4% paraformaldehyde for 30 min and washed twice with PBS for 2 min. Fixed cells in permeabilization solution (0.1%Triton X-100 and 0.1% Sodium citrate) were washed and incubated with TUNEL assay mixture for 60 min. The TUNEL-stained cells were visualized by a FLUOVIEW FV10i confocal microscopy (Olympus, Tokyo, Japan).

### 2.5. Cell Cycle Analysis for Sub-G1 Accumulation

Cell cycle analysis was performed by propidium iodide (PI) staining. SW480 or HCT116 cells treated with EGCG and/or TRAIL for 24 h were harvested and fixed in 70% ethanol. The cells were then incubated at 37 °C with 0.1% ribonuclease A in PBS for 30 min and suspended in PBS containing 30 μg/mL PI for 30 min at room temperature. Sub-G1 accumulation was evaluated from the stained cells by FACS Calibur (Becton Dickinson, Franklin Lakes, NJ USA) using the Cell Quest program (Becton Dickinson, Franklin Lakes, NJ USA).

### 2.6. FACS Analysis for Early and Late Apoptosis Detection

SW480 or HCT116 cells (2 × 10^5^ cells/well) in six-well plates were treated with EGCG and/or TRAIL for 24 h and stained with Annexin V-FITC and PI kit (Bio Vision Technology Inc., Golden, CO, USA). Then the cells were analyzed using Cell Quest Software with the FACS Calibur flow cytometer (Becton Dickinson, Franklin Lakes, NJ, USA).

### 2.7. FACS Analysis for Cell Surface Expression of DR5

To determine the surface expression of the DR5, FACS analysis was carried out in SW480 and HCT116 cells treated with EGCG and/or TRAIL for 24 h were twice washed with PBS and incubated with 10 μg/mL DR5-FITC conjugated or mouse IgG antibody (Abcam, United Kingdom) in PBS for 1 h at 4 °C. After washing, cells were analyzed by flow cytometry using a FACS Calibur flow cytometer (Becton Dickinson, Franklin Lakes, NJ, USA).

### 2.8. RNA Isolation and Measurement

Total RNAs from SW480 and HCT116 cells treated with EGCG and/or TRAIL were isolated by using the Trizol reagent (Life technologies, Inc., Carlsbad, CA, USA) according to the manufacturer’s instructions. In brief, each sample was lysed in the Trizol reagent by repeated pipetting and then 0.2 mL chloroform per 1 mL Trizol reagent was added, vortexing vigorously. After the mixture was centrifuged at 12,000× *g* for 15 min at 4 °C, the mixture was separated into the colorless upper aqueous phase, a lower red, phenol-chloroform phase and interphase. The RNAs were selectively precipitated from aqueous phase by mixing with 0.5 mL isopropanol and washed with 75% EtOH. The dried RNA pellets were dissolved with RNase-free water and the eluted RNA was quantified using a ND-1000 spectrophotometer (NanoDrop Technologies, Inc., Wilmington, DE). The quantity of the RNAs was verified by 1% agarose denaturing gel and an Agilent 2100 bio-analyzer (Agilent Technologies, Palo Alto, CA, USA).

### 2.9. Quantitative Real Time Polymerase Chain Reaction (qRT-PCR)

From total RNAs isolated from SW480 and HCT116 cells treated with EGCG and/or TRAIL, one microgram of total RNA was used to make cDNA by Superscript reverse transcriptase and amplified by Platinum Taq polymerase with Superscript One Step RT-PCR kit (Invitrogen, Carsbad, CA, USA). Primers sequences were synthesized by Bioneer (Daejeon, Korea) with the following sequences: hDR5- forward: 5’- GAC TCT GAG ACA GTG CTT CGA TGA -3’; reverse- 5’-CCA TGA GGC CCA ACT TCC T-3’, hGAPDH-forward5’-CCA CTC CTC CAC CTT TGA CA-3’; reverse-5’-ACC CTG TTG CTG TAG CCA -3’. For PCR amplification, the following steps were undertaken; an initial step at 50 °C for 30 min, 94 °C for 2 min, followed by 30 cycles at 94 °C for 15 s, 55 °C for 30 s and 72 °C for 1 min, and a final step at 72 °C for 10 min. The amplified products were separated on 2% agarose gel. Then RT-qPCR was performed with the LightCycler TM instrument (Roche Applied Sciences, Indianapolis, IN, USA).

### 2.10. Western Blotting

SW480 or HCT116 cells were exposed to EGCG and/or TRAIL for 24 h and were lysed in RIPA buffer (50 mM Tris-HCl, 150 mM NaCl, 2 mM EDTA and 1% Triton X-100) containing protease inhibitors and phosphatase inhibitors. The protein samples were separated on 8% to 15% sodium dodecyl sulfate-polyacrylamide gels (SDS-PAGE) and were transferred to nitrocellulose membranes. Membranes were incubated with primary antibodies of PARP, Cleaved PARP, caspase 8 (Cell Signaling, Beverly, MA, USA), DR5, DR4 (Santa Cruz Biotechnology, CA, USA) and β-actin (Sigma Aldrich Co., St. Louis, MO, USA). These were diluted in 3% bovine serum albumin (BSA) and in PBS-Tween 20 (1:500–1:2000) at 4 °C overnight, washed three times with PBS-Tween20 and finally incubated with HRP-conjugated secondary antibody (1:2000) in 3% skim milk. The expression was visualized by using ECL Western blotting detection reagent (GE Healthcare, Amersham, UK).

### 2.11. RNA Interference

SW480 or HCT116 cells were transfected with scrambled small interfering RNA (siRNA) or DR5 siRNA plasmid (Bioneer, Korea) with Interferin™ transfection reagent (Polyplus-transfection Inc., New York, NY, USA). The mixtures of DR5 siRNA (40 nM) and Interferin™ transfection reagent were incubated for 15 min, and then, the cells were incubated at 37 °C for 48 h before exposure to EGCG and/or TRAIL for 24 h.

### 2.12. Statistical Analysis

The data were expressed as means ± SD from at least three independent experiments. Student’s t-test was used for two group comparison. In addition, the one-way analysis of variance (ANOVA) followed by a Turkey post-hoc test was applied for multi-group comparison using GraphPad Prism software (Version 5.0, California, USA). The difference between groups was considered statistically significant, if the ***p***-value was less than 0.05.

## 3. Results

### 3.1. Cotreatment of TRAIL and EGCG Synergistically Increased Cytotoxicity in CRCs

**Figure 1:**
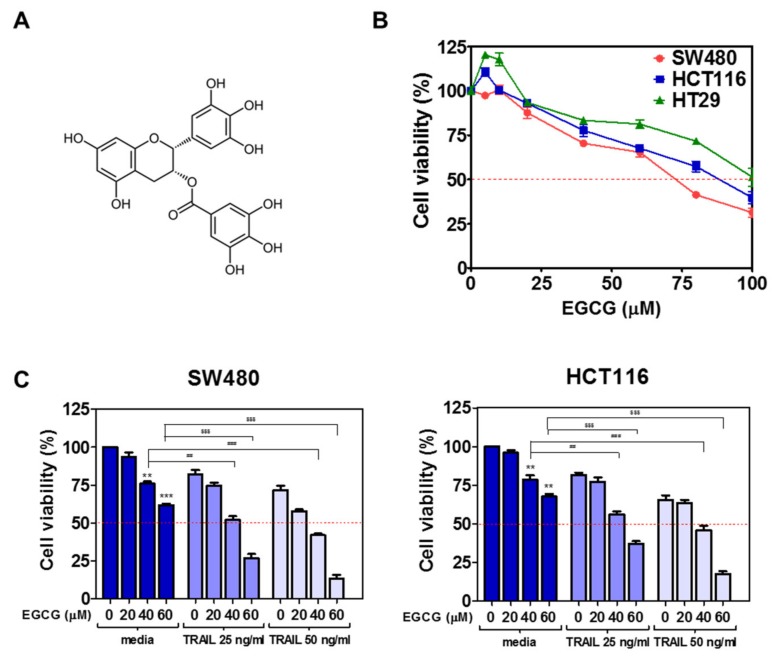
Synergistic cytotoxic effect of epigallocatechin-3-gallate (EGCG) and tumor-necrosis-factor-related apoptosis-inducing ligand (TRAIL) cotreatment in colorectal cancer cells. (**A**) Chemical structure of EGCG. Molecular weight = 458.37. (**B**) SW480, HCT116 and HT29 cells were seeded into 96-well microplates and treated with various concentrations (5, 10, 20, 40, 60, 80, 100 µM) of EGCG for 24 h. Cell viability was measured by 3-(4,5-dimethylthiazol-2-yl)-2,5-diphenyltetrazolium bromide (MTT) assay. (**C**) Synergistic effect of EGCG on the cytotoxicity of TRAIL in SW480 and HCT116 cells. Two colon cancer cell lines were treated with the indicated concentrations of EGCG and/or TRAIL for 24 h. Then cell viability was analyzed using MTT assay. ** *p* < 0.01, *** *p* < 0.001 vs. untreated control, ## *p* <0.01, ### *p* < 0.001 vs. TRAIL (25 or 50 ng/mL) at 40 µM EGCGl, $$$ p < 0.001 vs. TRAIL (25 or 50 ng/mL) at 60 µM EGCG

To examine the effect of EGCG (Figure 1A) against colon cancer cells, MTT assay was performed. To assess the cytotoxicity of EGCG and/or TRAIL, MTT assay was conducted in HT29, SW480, HCT116 cells. To assess the synergy of EGCG and TRAIL, SW480 and HCT116 cells were treated by various concentrations of EGCG (0, 20, 40, 60 μM), and/ or TRAIL (0, 25, 50 ng) for 24 h. The concentrations of TRAIL alone were decided based on Kim et al.’s paper that suggested TRAIL showed sensitivity to HCT116 cells in a concentration dependent manner [19]. As shown in Figure 1B, cotreatment of EGCG and TRAIL showed synergistic cytotoxicity in three CRCs. Furthermore, the combination index (CI) values were found less than 0.5 at all fraction-affected points (Figure 1C), indicating evident synergy of TRAIL and EGCG in cytotoxicity.

### 3.2. Cotreatment of EGCG and TRAIL Dramatically Increased TUNEL-Positive Cells, Cleaved PARP and Activated Caspase 8 in SW480 and HCT116 Cells

**Figure 2:**
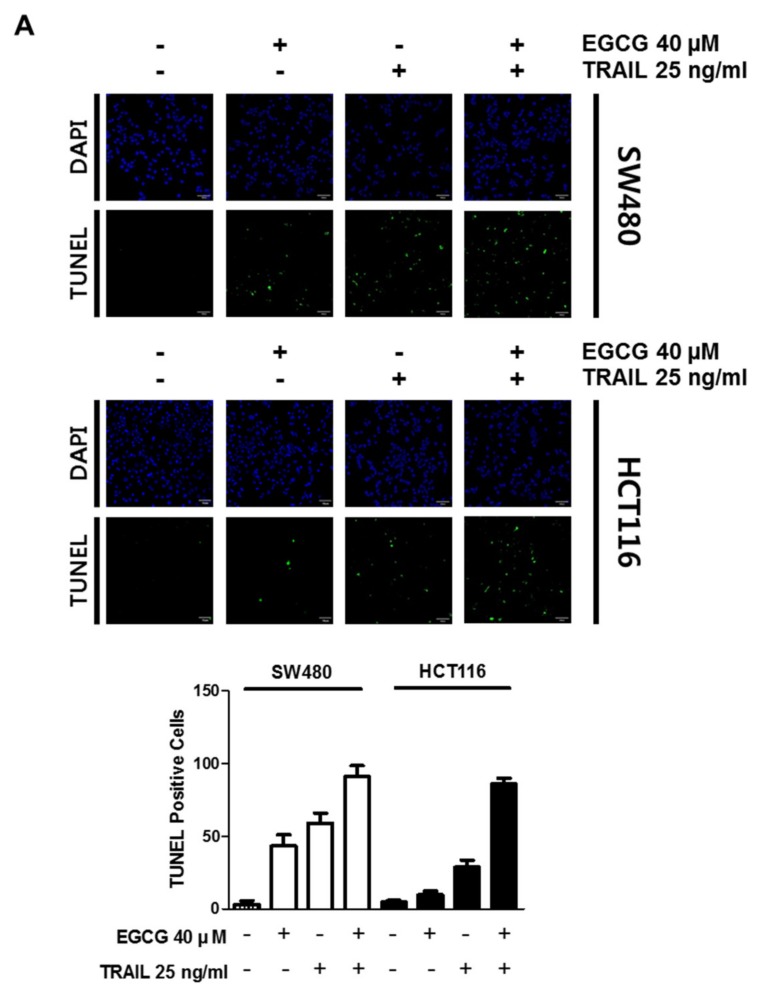

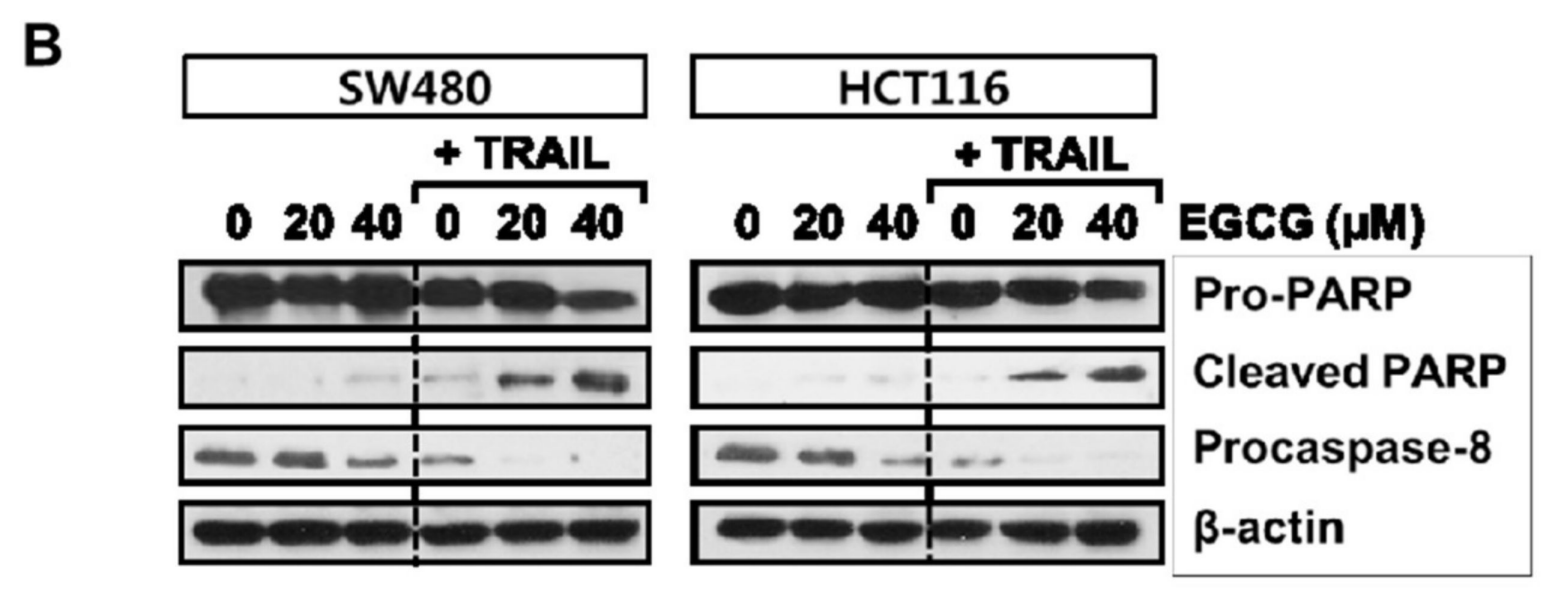
Cotreatment of EGCG and TRAIL increased the number of terminal deoxynucleotidyl transferase-dT-mediated dUTP nick end labelling (TUNEL)-positive cells and induced poly (adenosine diphosphate-ribose) polymerase (PARP) cleavage and caspase 8 activation in SW480 and HCT116 cells. (**A**) Effect of EGCG and TRAIL on the number of TUNEL-positive cells in SW480 and HCT116 cells. SW480 and HCT116 cells were treated with TRAIL 25 ng/mL and/or EGCG 40 µM for TUNEL staining. The fluorescent signals from fragmented DNA (green), and DAPI (blue) were visualized and photographed by FLUOVIEW FV10i confocal microscopy. Magnification bar = 50 µm. Bar graphs represent quantification of TUNEL-positive cells (%). (**B**) Effect of TRAIL and EGCG on PARP cleavage and caspase 8 in in SW480 and HCT116 cells. SW480 and HCT116 cells were treated with TRAIL (25 ng/mL) and/or EGCG (20, 40 μM) for 24 h. Cell lysates were prepared and subjected to Western blotting with the indicated antibodies. β-actin was used as an internal control.

To assess whether the cytotoxicity by cotreatment of EGCG and TRAIL is exerted by apoptosis, TUNEL assay and Western blotting were conducted. Here, TUNEL assay revealed that EGCG and TRAIL cotreatment significantly increased the number of TUNEL-positive cells in SW480 and HCT116 cells (Figure 2A). Consistently, cotreatment of TRAIL and/or EGCG significantly attenuated the expression of procaspase 8 and cleaved PARP at protein level in SW480 and HCT116 cells (Figure 2B).

### 3.3. Cotreatment of EGCG and TRAIL Upregulated DR5 in Colon Cancer Cells

**Figure 3:**
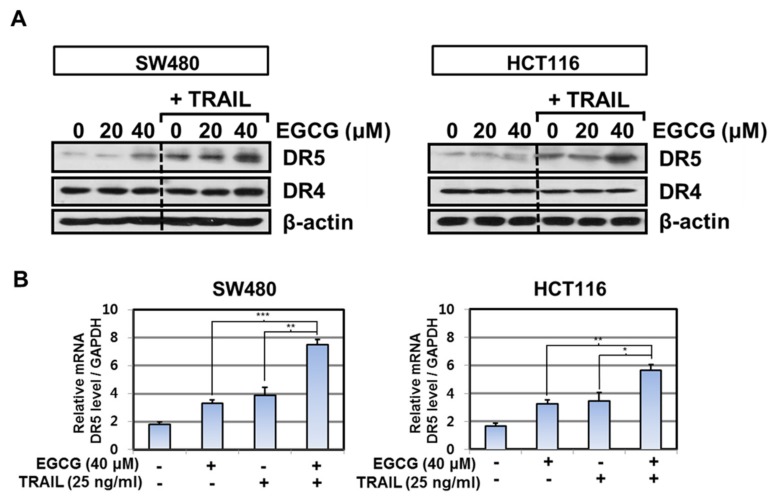

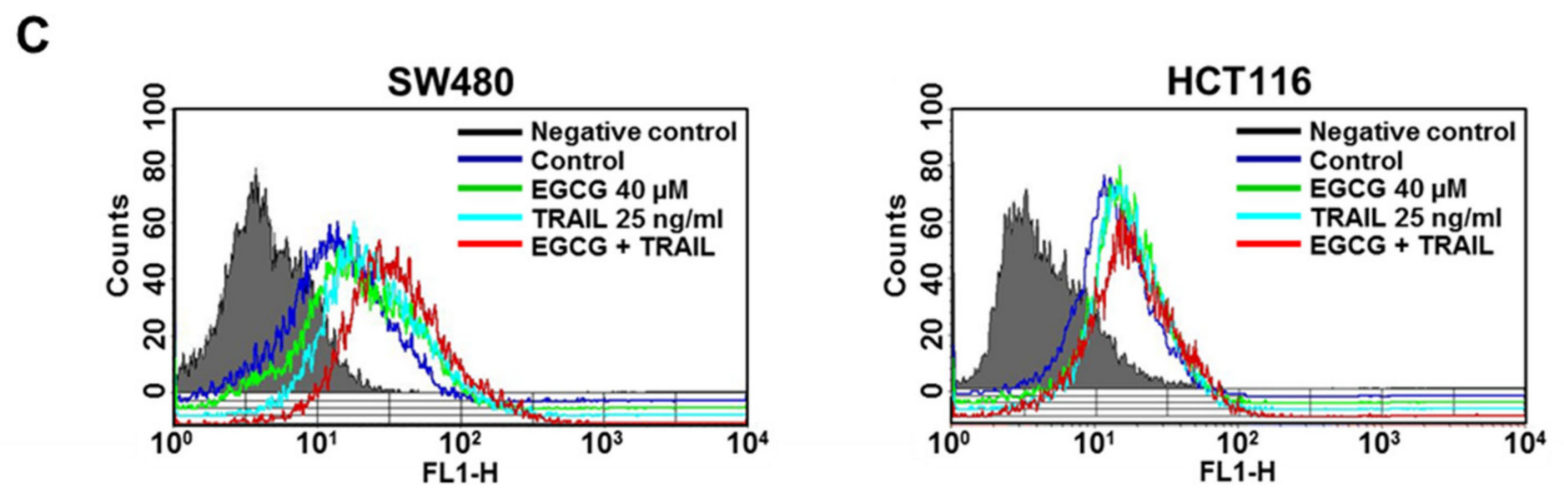
Effect of EGCG and TRAIL cotreatment on the expression of death receptor 5 (DR5) in SW480 and HCT116 cells. (**A**) SW480 and HCT116 cells were treated with TRAIL (25 ng/mL) and/or EGCG (20, 40 μM) for 24 h. Cell extracts were prepared and subjected to Western blotting with death receptor 4 (DR4) and DR5 antibodies. β-actin was used as an internal control. (**B**) SW480 or HCT116 cells were treated with 40 μM EGCG and 25 ng/mL TRAIL. Total RNA was isolated from the treated cells, and expression of DR5 at mRNA level was determined by RT-qPCR. The data were analyzed by two-way analysis of variance. * p < 0.05, ** p < 0.01, ***p < 0.001 vs. co-treated EGCG 40 µM and TRAIL 25 ng/mL. (**C**) SW480 and HCT116 cells were treated with 40 µM EGCG and/or TRAIL 25 ng/mL for 24 h. Cells were then stained with FITC-conjugated antibodies for DR5 or IgG control (negative control), and expression of DR5 on the cell surface was analyzed using flow cytometry.

It is well documented that TRAIL resistance in cancers is attributed to downregulation of the upregulation of decoy receptors (DcR1 and DcR2) and/or death receptors (DR4 or/and DR5) [20,21]. Thus, the effect of EGCG and/or TRAIL was evaluated on the expression of death receptor-related genes such as DR4 or DR5 in SW480 and HCT116 cells. Herein, Western blotting revealed that cotreatment of EGCG and TRAIL enhanced the expression of DR5 at protein level but not DR4 (Figure 3A). Consistently, cotreatment of EGCG and TRAIL increased the mRNA expression of DR5 in SW480 and HCT116 cells by qRT-PCR (Figure 3B). However, the cell surface DR5 expression was much more detected by cotreatment of EGCG and TRAIL compared to EGCG or TRAIL alone in SW480 cells, but not in HCT116 cells (Figure 3C).

### 3.4. Depletion of DR5 Reduced the Ability of EGCG and TRAIL Cotreatment to Induce Cytotoxicity, Cleaved-PARP and Increase Sub G1 Population in Colon Cancer Cells

**Figure 4:**
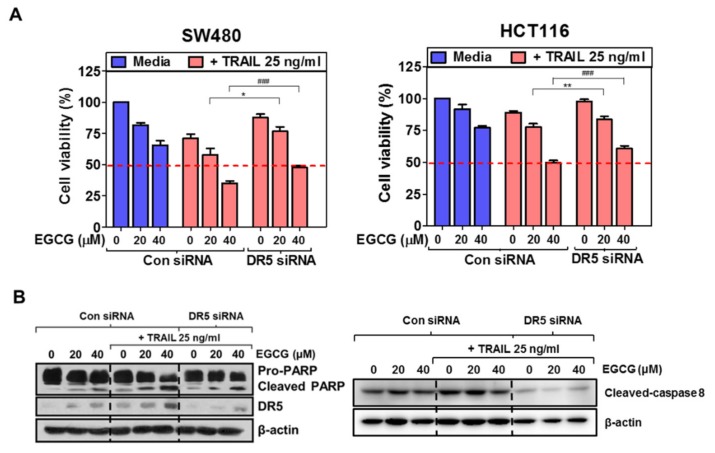

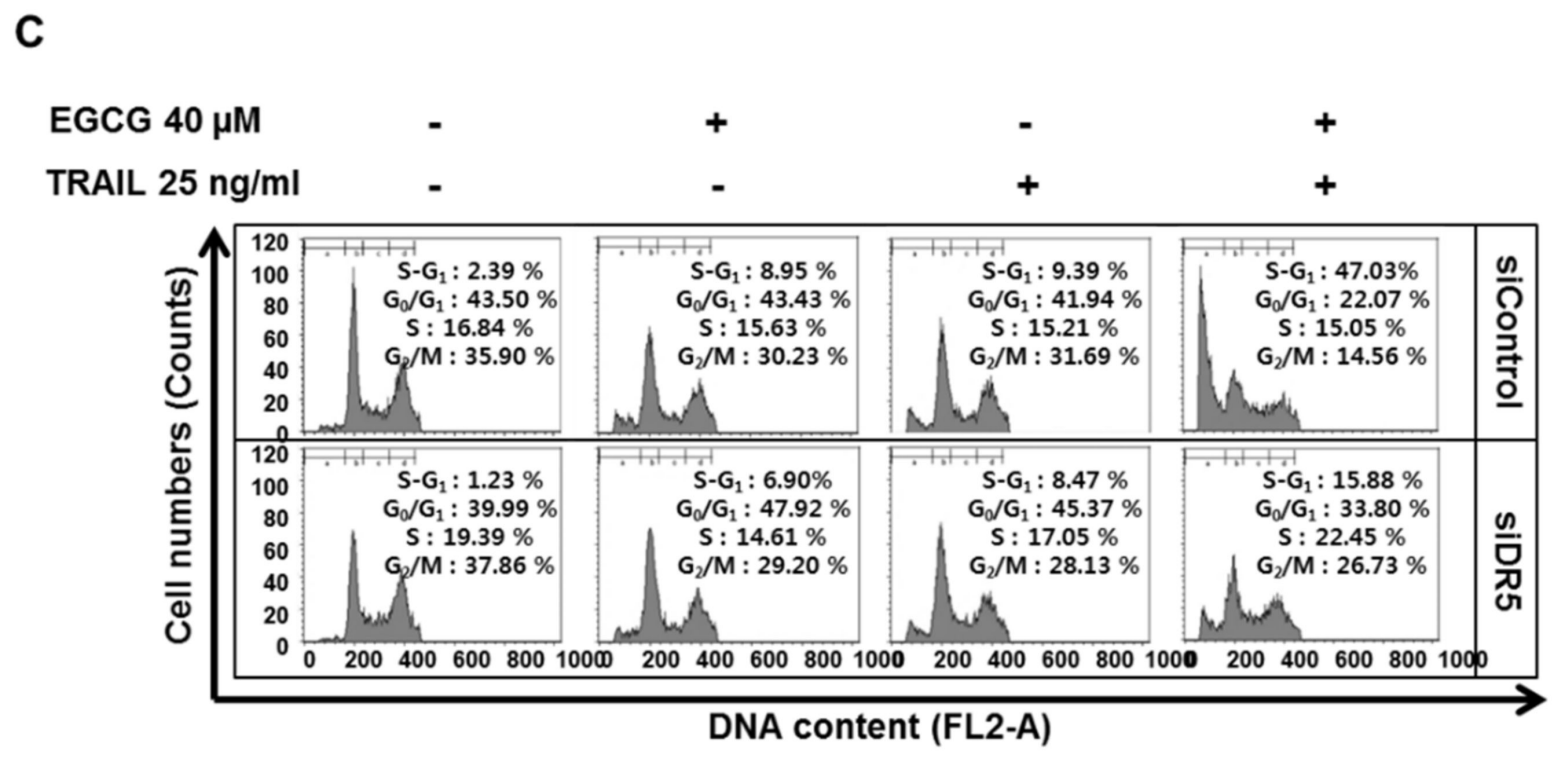
Depletion of DR5 blocks apoptosis induced by EGCG and TRAIL cotreatment in SW480 and HCT116 cells. (**A**) SW480 and HCT 116 cells were transiently transfected with control siRNA or DR5 siRNA plasmids and exposed to TRAIL (25 ng/ml) and EGCG (20 and 40 µM) for 24 h. Cell viability was measured by MTT assay. * *p* < 0.05, ** *p* < 0.01 vs. DR5 siRNA control at 20 µM of EGCG. ### *p* < 0.01, vs. DR5 siRNA control at 40 µM of EGCG. (**B**) SW480 cells were transfected with DR5 siRNA plasmid and treated with EGCG and/or TRAIL for 24 h. The lysates were subject to Western blotting for PARP, DR5 and β-actin. (**C**) Effect of DR5 silencing on sub-G1 population by cotreatment of EGCG and TRAIL in SW480 cells. FACS analysis was conducted for sub-G1 accumulation in DR5-depleted SW480 and HCT116 cells following exposure to cotreatment of EGCG and TRAIL for 24 h/mL. Redline indicates IC50.

To examine whether DR5 upregulation is critically involved in the sensitizing effect of EGCG and/or TRAIL of SW480 and HCT116 cells, SW480 and HCT116 cells transfected with control or DR5 siRNA plasmids with or without treatment of EGCG and/or TRAIL were subjected to MTT assay, Western blotting and cell cycle analysis. The cytotoxicity by cotreatment of EGCG and TRAIL was significantly reduced in DR5-depleted SW480 and HCT116 cells (Figure 4A). Consistently, DR5 depletion suppressed the ability of EGCG and TRAIL cotreatment to cleave PARP in SW480 and HCT116 cells (Figure 4B). Similarly, DR5 depletion decreased sub-G1 population to 15.88% compared to untreated control (47.3%) in EGCG and TRAIL cotreated SW480 and HCT116 cells (Figure 4C).

## 4. Discussion

Though TRAIL has been used as a potent anticancer agent in several cancers such as prostate [20], lung cancers [21], breast [22] and ovarian [23], its resistance is an important issue during cancer therapy. Thus, TRAIL-based cancer therapy is one of the most promising therapeutic strategies. Recently, natural compounds were reported to enhance apoptosis by their cotreatment with TRAIL, targeting DR5 [24,25]. Indeed, curcumin [26,27], tanshinone I [28], tanshinone IIA [29], propolis [30,31] and kaempferol [32] have been attractive as TRAIL sensitizers in several cancers. In the same line, present work provides scientific evidence that EGCG sensitizes colon cancer cells to TRAIL induced apoptosis via caspase 8 and DR5 signaling.

The dysregulation of apoptosis causes several diseases, including cancer. Apoptosis is classified mainly into extrinsic pathway (cell death receptor dependent) and intrinsic pathway (mitochondrial dependent) [33,34,35]. During the process of apoptosis, PARP is usually detected for DNA breakage by cleavage of NAD+ into nicotinamide and ADP-ribose [36]. Herein cotreatment of EGCG and TRAIL revealed synergistic cytotoxicity in SW480 and HCT116 cells compared to EGCG or TRAIL alone. Moreover, cotreatment EGCG and TRAIL cleaved PARP, activated caspase 8, increased the number of TUNEL-positive cells and increased sub G1 population as apoptotic features in SW480 and HCT116 cells, implying the synergistic apoptosis by EGCG and TRAIL cotreatment in CRCs.

To overcome the resistance of cancer cells to TRAIL, a lot of approaches have been tried including upregulation of death receptor DR4 and/or DR5 [37,38]. Our Western blotting revealed that cotreatment of EGCG and TRAIL activated caspase 8 and DR5 in SW480 and HCT116 cells and significantly increased cell surface DR5 expression in SW480, but not in HCT116 cells by FACS analysis. Conversely, depletion of DR5 using siRNA transfection reduced significant cytotoxicity by EGCG and TRAIL co-treatment in in SW480 and HCT116 cells and also blocked the apoptotic effects of EGCG and TRAIL co-treatment to increase sub-G1 population and PARP cleavages in SW480 cells, indicating that EGCG sensitizes colon cancer cells to TRAIL mediated apoptosis via activation of caspase 8 and upregulation of DR5. Similarly, Kim et al. reported that Parthenolide sensitizes TRAIL-induced apoptosis via upregulation of DR5 and mitochondria-dependent pathway.

## 5. Conclusions

In summary, cotreatment of EGCG and TRAIL enhanced synergistic cytotoxicity, early and late apoptotic portion, sub G1 accumulation, the number of TUNEL-positive cells, potentiated the PARP cleavage and increased caspase 8 activation compared to TRAIL or EGCG alone in SW480 and HCT116 cells. Furthermore, co-treatment of EGCG and TRAIL enhanced the expression of DR5 at protein and mRNA levels. Conversely, DR5 depletion blocked the apoptotic effects of cotreatment of EGCG and TRAIL to increase cytotoxicity, sub-G1 accumulation and PARP cleavages in SW480 and HCT116 cells. Taken together, these findings suggest that EGCG can be a potent TRAIL sensitizer via extrinsic apoptotic pathway such as caspase 8 and DR5 activation in colorectal cancer cells [19].
